# The use of antibiotic-loaded bone cement does not increase antibiotic resistance after primary total joint arthroplasty

**DOI:** 10.1007/s00167-021-06649-x

**Published:** 2021-07-09

**Authors:** Kaspar Tootsi, Victoria Heesen, Martin Lohrengel, Andreas Eugen Enz, Sebastian Illiger, Wolfram Mittelmeier, Christoph H. Lohmann

**Affiliations:** 1 Department of Orthopaedics, Otto-Von-Guericke University, Leipziger Str. 44, 39120 Magdeburg, Germany; 2 Department of Traumatology and Orthopaedics, University of Tartu, Puusepa Str 8, 51014 Tartu, Estonia; 3 Department of Orthopaedics, Tartu University Hospital, Puusepa Str 8, 51014 Tartu, Estonia; 4 Klinik und Poliklinik für Orthopädie, Universitätsmedizin Rostock, Doberaner Straße 142, 18057 Rostock, Germany

**Keywords:** Antimicrobial resistance, Antibiotic-loaded cement, Arthroplasty, Periprosthetic joint infection, Gentamicin, Clindamycin

## Abstract

**Purpose:**

One of the preventive strategies for periprosthetic joint infection (PJI) is the use of antibiotic-loaded bone cement (ALBC) in primary total joint arthroplasty (TJA). Even though it is widely used, there are concerns about the development of antibacterial resistance. The aim of the study was to investigate whether using ALBC in primary TJA increases the antibiotic-resistant PJI. The hypothesis was that the regular use of ALBC does not increase the rate of resistant PJI.

**Methods:**

Patients with confirmed PJI who had revision surgery from year 2010 to 2019 were included in this international multicenter study. The ALBC group was compared to the non-ALBC TJA group from the same time period. Medical records were used to collect clinical (age, gender, body mass index, comorbidities), TJA-related (type of operation, implant type and survival) and PJI-related (cultured microorganism, antibiogram) data. Resistance to gentamicin, clindamycin and vancomycin were recorded from the antibiograms. Multiple logistic regression model was used to identify risk factors and account for the potential confounders.

**Results:**

218 patients with PJI were included in the study: 142 with gentamicin-loaded bone cement and 76 in the non-ALBC group. The average age in the ALBC group was 71 ± 10 years and 62 ± 12 years in the comparison group (*p* < 0.001). Coagulase negative Staphylococci (CONS) were the most common (49%) isolated pathogens. The use of ALBC did not increase the rate of any resistant bacteria significantly (OR = 0.79 (0.42–1.48), *p* = 0.469). The presence of CONS was associated with higher risk of antibiotic resistance.

**Conclusions:**

The current study demonstrates no increase in antibiotic resistance due to ALBC after primary TJA. Thus, the use of ALBC during primary TJA should not be feared in the context of antimicrobial resistance.

**Level of evidence:**

III.

## Introduction

The widespread use of ALBC in various countries has raised concerns about the development of antibiotic resistance in patients with PJI [2, 29, 32]. Two different levels of antibiotics in the cement are differentiated: a low dose (< 2 g in 40 g of cement) for prophylactic use in primary arthroplasty and high concentration for infection treatment [5]. The prophylactic concentration of antibiotics in ALBC has been shown to be inconsequential to the mechanical properties of polymethylmethacrylate (PMMA) bone cement [13]. The use of ALBC enables to achieve high levels of antibiotics locally, while causing less systemic side-effect. Regardless of the benefits, several concerns have been raised for using ALBC as a routine practice that include potential increase in antibiotic resistance and decreased mechanical strength of bone cement [3, 9]. Unfortunately, clinical research investigating the benefits and risks of ALBC are limited and have failed to reach a uniform conclusion, therefore, the routine clinical practice varies hugely [31]. Several studies have shown decreased rates of surgical site infection for using ALBC, which is why it is a widely spread routine clinical practice, especially in Europe [10, 17, 35].

The growth of drug-resistant bacteria in PJI is concerning and the drastic rise of overall prevalence of PJI calls for interventions lowering the infection rate [2, 22]. The use of ALBC requires specific attention for determining the long-term risk of inducing antibacterial resistance. Hope et al. have published an increased prevalence up to 30% of gentamicin resistant bacteria in revision TJA after prior exposure to ALBC [15]. Other larger analysis found no significant difference [12]. Therefore, the data are limited, based on small single-center studies and contradictory, thus larger studies including data from multinational centers is highly needed.

The aim of the present study was to determine whether the use of ALBC is associated with higher prevalence of antibiotic-resistant bacteria as a cause of PJI after primary total knee or hip joint replacement. Even low dose of antibiotics can potentially cause resistance due to not eradicating all of the bacteria and enabling the bacteria to develop mechanisms to counteract the antibiotics. The hypothesis of the present study was that using ALBC in primary TJA does not increase antibacterial resistance. Therefore, the first revisions of cemented or cementless primary total hip arthroplasties (THAs) or total knee arthroplasties (TKAs) were analyzed for occurrence of resistant bacteria in an international multicenter study. Thorough information about the bacterial susceptibility to clinically most relevant antibiotics (gentamicin, clindamycin and vancomycin) was gathered and analyzed.

## Methods

This multicenter study includes patients presenting with a knee or hip joint PJI. The study participants were recruited retrospectively from three university hospitals. Prerequisite for inclusion was no intraarticular surgery of the respective joint prior to the index surgery. A total of 218 patients met the inclusion and exclusion criteria during the study period and were included in the analysis. 62% of the study participants had their primary operation in one of the three study centers and the remaining 38% were initially operated somewhere else.

The study includes patients with confirmed PJI (≥ 2 positive cultures) who underwent revision surgery from December 2010 to December 2019. The resistograms were analyzed for any resistant bacteria, difficult to treat bacteria, particularly for resistance to gentamicin, since it was the only antibiotic added to the bone cement during primary TJA in these hospitals in the study period. The data were acquired from the hospital’s local database.

Patients with no prior implant-associated infection who were eligible for revision surgery were included in the study. Furthermore, only patients having at least two positive microbiological cultures with antibiogram were included.

Patients were excluded from the study if they had had any intraarticular surgery prior to the primary TJA in the respective joint or prior surgical revision of the respective joint. If a sepsis was documented prior to revision surgery or if there was missing documentation of the used bone cement, the patients were also excluded.

The antibiotic agent used in the ALBC group was gentamicin with different premixed formulations: Palacos R + G (0.5 g of gentamicin in 40 g of bone cement) (Heraeus Medical GmbH, Germany), Cemex Genta (1.0 g/40 g) (Merete GmbH, Germany), Optipac Refobacin R (0.5 g/40 g) and Biomet R (0 g/40) (Zimmer Biomet Holdings, Inc., USA), Smartset GHV (1.0 g/40 g) and Depuy CMW (1 g/40 g) (DePuy Synthes, Johnson & Johnson Corporation, USA). The comparison group comprised of cementless and cemented without the ALBC PJI revision cases from the same three hospitals. The same inclusion criteria applied for the non-ALBC group, except for the use of ALBC during the primary TJA.

During revision surgery, at least five tissue cultures were obtained. Tissues were transferred to the hospital’s Department of Microbiology within 30 min after retrieving the tissue samples from the infected joints to ensure diagnostic quality, particularly with respect to anaerobic bacteria. The resistograms and specific antimicrobial resistance of the bacteria were obtained after culture for 2 weeks. Resistance to gentamicin, clindamycin and vancomycin were assessed according to the EUCAST guidelines [18]. Proven PJI was considered when at least two samples were positive for bacteria.

Early PJI was defined as symptom development within the first 6 weeks from the primary TJA and symptom development > 6 weeks after implantation was defined as a late infection. Charlson comorbidity index and American Society of Anaesthesiologists (ASA) score was used to describe the comorbidities within the study groups [7]. Survival of the implants was measured from the primary TJA to the revision surgery in years.

The study has been approved by the local Ethics Committees (Otto von Guericke University, No 106/17; Rostock University Medical Center, No A2020-0297; University of Tartu, No 330/T-20).

### Statistical analysis

Data are presented as mean ± SD. The Chi-square test or Fischer’s exact test were used to compare group proportions when appropriate. Student’s *t* test or Mann–Whitney *U* test were used for group comparisons depending on the normality of the distribution of the data. Logistic regression analysis was used to include the potential confounding factors in the analysis. Statistical analysis of the data was performed by SPSS 26.0 (SPSS Inc., Chicago IL, USA) for Windows.

## Results

The general parameters of the study population are presented in Table 1. There were 142 participants in the ALBC group and 76 in the non-ALBC. The study includes 50 early and 168 late infections. The proportion of early infections, average implant survival and body mass index (BMI) did not differ significantly between the groups (Table 1). The type of primary TJA and the implant are presented in Tables 2 and 3, respectively.

**Table 1:** General parameters of antibiotic-loaded cement (ALBC) group and the non-ALBC group with periprosthetic joint infection

Variable	ALBC (*n* = 142)	Non-ALBC (*n* = 76)	*p* value
Age (years)	71 ± 10	62 ± 12	< 0.001
Male/female	72/70	54/22	0.006
THA/TKA (*n*)	50/92	64/12	< 0.001
BMI (kg/m^2^)	31.3 ± 6.5	30.3 ± 6.6	0.250
ASA score	2.7 ± 0.5	2.4 ± 0.5	0.005
Charlson comorbidity index	4.9 ± 2.9	3.4 ± 2.3	< 0.001
Early/late infections (*n*)	32/110	18/58	0.866
Implant survival (years)	3.7 ± 5.6	4.9 ± 9.8	0.303
Primary implantation year (mean (range))	2012 (1984–2019)	2012 (1993–2019)	0.250

*ALBC* antibiotic-loaded bone cement, *THA* total hip arthroplasty,* TKA* total knee arthroplasty, *BMI* body mass index, *ASA* American Society of Anaesthesiologists

**Table 2:** Type of fixation of primary implant

	*n*	Percent
Cementless THA	59	27.1
Hybrid THA	23	10.6
Cemented THA	32	14.7
Cementless TKA	9	4.1
Hybrid TKA	13	6.0
Cemented TKA	82	37.6
Total	218	100

*THA* total hip arthroplasty, *TKA* total knee arthroplasty

**Table 3:** Type of primary implant

	*n*	Percent
Standard THA	110	50.5
Short hip stem	1	0.5
Surface replacement THA	1	0.5
Proximal femoral replacement	2	0.9
Surface replacement TKA	94	43.1
Hinged TKA	8	3.7
Distal femoral replacement	2	0.9
Total	218	100

*THA* total hip arthroplasty, *TKA* total knee arthroplasty

Approximately half of the infections (48.6%) were caused by coagulase negative *Staphylococci* (CONS) (Table 4). The second most prevalent isolated pathogen was *Staphylococcus aureus* in 34.4% of the cases*.* The prevalence of the different isolated bacteria is presented in Table 4. Polymicrobial infection was identified in 16% of the study participants. The overall prevalence of bacteria with any recorded antibiotic resistance was 36.5% (76 of the 218 patients).

**Table 4:** List and proportion of pathogens isolated from periprosthetic joint infections

Isolated pathogen	ALBC group (*n*)	Non-ALBC group (*n*)	Total (%)
CONS	71	35	48.6
* Staphylococcus epidermidis*	50	23	33.5
* Staphylococcus capitis*	3	5	3.7
* Staphylococcus hominis*	4	2	2.8
* Staphylococcus lugdunensis*	3	2	2.3
* Staphylococcus warneri*	2	1	1.4
* Staphylococcus haemolyticus*	2	1	1.8
* Staphylococcus pasteuri*	1	0	0.5
* Staphylococcus caprae*	2	0	0.9
* Staphylococcus simulans*	2	0	0.9
* Staphylococcus vitulinus*	0	1	0.5
* Staphylococcus oralis*	1	0	0.5
*Staphylococcus aureus*	49	26	34.4
*Enterococcus faecalis*	11	4	6.9
*Propionibacterium acnes/avidum*	1	5	2.8
*Escherichia coli*	2	4	2.8
*Pseudomonas aeruginosa*	13	0	1.4
*Bacteroides fragilis*	0	1	0.5
*Finegoldia magna*	1	0	0.5
*Corynebacterium amycolatum*	1	0	0.5
*Micrococcus luteus*	1	0	0.5
*Cornybacterium tuberculostearium*	0	1	0.5
*Trueperella bernardiae*	1	0	0.5
*Streptococcus agalactiae*	4	2	2.8
*Bacillus spp*	0	4	1.8
*Pseudomonas stutzeri*	1	0	0.5
*Streptococcus pyogenes*	1	1	0.9
*Bacillus pumilus*	0	1	0.5
*Citrobacter koseri*	0	1	0.5
*Acinetobacter haemolyticus*	0	1	0.5
*Bacillus cereus*	0	1	0.5
*Acinetobacter schindleri*	1	0	0.5
*Streptococcus dysgalactiae*	4	4	3.7
*Streptococcus anginosus*	1	1	0.9
*Streptococcus salivarius*	1	0	0.5
*Candida parapsi*	1	0	0.5
*Enterobacter cloacae*	1	0	0.5
*Listeria monocytogenes*	1	0	0.5
*Streptococcus gordonii*	1	0	0.5
*Parvimonas micra*	1	0	0.5

*CONS* coagulase negative *Staphylococci*

Table 5 demonstrates the proportions of resistant bacteria in each study group. There was no significant correlation for the use of ALBC and having any resistant bacteria (*p* = 0.658). Also, there were no significant differences for gentamicin or clindamycin resistance between the study groups.

**Table 5:** Proportions of antibiotic resistance in the antibiotic-loaded bone cement and in the non-ALBC arthroplasty groups

	ALBC (*n* = 142)	Non-ALBC (*n* = 76)	*p* value
Any resistant bacteria (*n* (%))	48 (34)	28 (37)	0.658
Resistant bacteria to gentamicin (*n* (%))	27 (19)	7 (9)	0.076
Resistant bacteria to clindamycin (*n* (%))	26 (19)	18 (24)	0.381
Resistant bacteria to vancomycin (*n* (%))	3 (2)	1 (1)	0.860

*ALBC* antibiotic-loaded bone cement

To identify variables associated with the antibiotic resistance and account for the differences in clinical parameters between the study groups, a logistic regression analysis was performed (Table 6). The model revealed that the use of ALBC in the primary TJA was not associated with the prevalence of resistant bacterial strains. However, the presence of CONS was significantly associated with antibiotic resistance. A model investigating the determinants of pure gentamicin resistance found no significant effect of the use of ALBC (data not shown).

**Table 6:** Logistic regression analysis showing no significant predictors for having antibiotic-resistant bacteria

	*B*	OR	CI (95%)	*p* value
ALBC	− 0.24	0.79	0.43–1.48	0.469
Charlson comorbidity index	0.03	1.03	0.92–1.15	0.573
Implant survival in years	− 0.06	0.94	0.88–1.00	0.060
Presence of CONS	0.90	2.46	1.38–4.39	0.002

*OR* odds ratio, *CI* confidence interval, *ALBC* antibiotic-loaded bone cement, *CONS* coagulase negative *Staphylococci*

## Discussion

The most important finding of the study was the absence of a significant increase of antibiotic resistance for using ALBC. In the present study, a group of 218 patients with PJI undergoing revision surgery was examined to determine whether the use of ALBC for primary TJA increased the rate of resistant bacteria. The results demonstrate no significant increase in the prevalence of resistant bacteria after using ALBC in the primary TJA. Interestingly, positive CONS culture was significantly associated with the prevalence of resistant strains.

PJI is among the most devastating complications of TJA that can lead to repeated surgeries, poor functional outcome and even amputation of the limb and death [19]. Even though the rate of PJI is low (< 1% in most of the high-volume arthroplasty centers), the steady increase in the absolute number of TJA leads to increase in PJI [6, 21]. Therefore, prevention of PJI is of utmost importance.

Different strategies have been applied to prevent PJI. The use of intravenous preoperative antibiotics is among the most effective approaches. Adding antibiotics to the bone cement in addition to intravenous use is an effective prevention method that has shown to decrease the PJI rate in several high-quality studies up to 50% [8, 10, 26]. Gentamicin and clindamycin are the most often added antibiotics to the bone cement [38]. Low-dose mixture (< 2 g per 40 g of bone cement) is used for prevention in primary TJA. ALBC has been shown to be associated with lower rate of PJI in primary TJA, however, it is still not a routine practice worldwide, because of higher cost and safety concerns [16].

Three main problems may be associated with the safety of routine use of ALBC: decreased mechanical strength of the cement, local and systemic toxicity, and induction of antibiotic-resistant bacterial strains. Nevertheless, low levels of added antibiotics to the ALBC that is optimal for primary TKA and THA have not been demonstrated to cause negative clinical consequences [13, 35]. Gentamicin levels of ≤ 1 g in 40 g of bone cement that are mostly used in primary TJA, do not affect the mechanical strength [9]. Also, the cytotoxicity of ALBC has not been established to be clinically relevant [35]. Therefore, currently the most important safety issue is the possible induction of antibiotic-resistant bacterial strains, which is the main focus of the present study.

The length of antibiotics release from ALBC is enough to cover the perioperative period of TJA, however, after several weeks, the concentration falls to subtherapeutic levels, which might promote the development of antibiotic resistance [11]. Elution characteristics of the ALBC may be different depending on effective joint space (enlarged in loosened TJA’s), implant surface area and different joints. However, the direct effects of eluted antibiotics from bone cements on bacteria follow similar principles and the initial burst release from the ALBC is sufficient to eradicate the bacteria and not cause resistance. In addition, the development of resistance might also be associated with the biomaterial properties of bone cement [1, 20, 36]. Several mechanisms have been described, how resistance to antibiotics is developed. De novo mutations of previously susceptible bacteria are most probably the leading cause of resistance [23]. The use of antibiotics will provide a competitive advantage to the resistant mutated bacteria. Also, the existing bacteria resistant to antibiotics may simply be selected out in an antibiotic rich environment [38]. Furthermore, resistance genes can be spread between different strains of bacteria through plasmids of chromosomal inserts (via bacteriophages and transposons) [23].

The present study demonstrated that using ALBC in primary TKA and THA did not increase the prevalence of antibiotic-resistant bacterial PJI in the present study’s cohort. This suggests that other factors than ALBC might be more importantly impacting the spread of antibiotic resistance. Interestingly, the presence of CONS was associated with increased rate of resistant bacteria (Table 6). CONS are part of normal skin flora that can cause infection in immunocompromised patients and in patients with large foreign bodies (e.g. arthroplasty implants). The spread of resistant CONS is promoted by the use of antibiotics in the hospitals that provides a reservoir-resistant bacteria. Even though the origin of the infectious bacteria is unknown, the hospitals are one of the most probable places.

Other possible factors include: (1) inappropriate prescription or application of antibiotics, (2) high and increasing prevalence of multi-drug-resistant bacteria in the hospital, (3) small number of new and effective antimicrobials to efficiently eradicate resistant strains, (4) widespread agricultural use of antibiotics leading natural selection towards resistant strains that are transferred to humans through food supply [24, 25, 34, 37].

The results of the present study indicating no increased antibiotic resistance due to ALBC are supported by several studies [9, 12]. However, some published data suggest a significantly higher prevalence of antibiotic-resistant bacteria with ALBC [8, 14, 33]. The largest analysis so far from the UK registry data found that the use of ALBC may increase the resistance to gentamicin [14]. However, that study only looked at Staphylococcal infections [14]. Even though the Staphylococci are the largest group as the cause of PJI, a sizable proportion of infections are caused by other microorganisms (33% in the current study) that were not included in the UK cohort. Thus, the present study provides a significant novel contribution by including detailed data about the whole spectrum of pathogens in PJI.

The most prevalent pathogens isolated from the PJI in the present study were CONS (mostly *S. epidermidis*) and *Staphylococcus aureus* that is consistent with previous research [2, 25, 27, 33]. However, the rate of polymicrobial infections is substantially higher (16%) in the present study compared with previously reported rates of 6% [33]. The difference might occur due to a genuine shift towards more pathogens in PJI, however, there are also some methodological considerations: the present study is more recent and the sample collecting and the microbiology culture methods might have changed during the time period (ca 10 years) to be able to detect more pathogens.

The present study has various limitations: the retrospective nature does not allow to confirm cause and effect. Other risk factors for developing resistance that were not included in the analysis might confound the effect of ALBC. Several different premixed ALBC-s with varying elution properties were used in the study that might have a confounding effect on the development of antibacterial resistance. Furthermore, the origin of the bacteria that caused the PJI is not known in the present study. The control group consists mainly of cementless TJA-s that might introduce a bias due to the absence of bone cement in the non-ALBC group. Nevertheless, the study population is thoroughly described compared with registry studies, all cultured microorganisms from the PJI samples have been included in the analysis, the bacteria have been identified on species level and the study entails resistance information about clinically most relevant antibiotics (gentamicin, clindamycin and vancomycin).

Current evidence together with the results of the present study suggest that the use of ALBC is a safe and effective method for preventing PJI and should be encouraged.

## Conclusion

The present study demonstrates that the standard use ALBC for the fixation of primary TJA does not increase the prevalence of antibacterial resistance. Having CONS as the causative agent of PJI is associated with increased risk for antibiotic resistance.
